# The Influence of Decontamination Procedures on the Surface of Two Polymeric Liners Used in Prosthodontics

**DOI:** 10.3390/polym13244340

**Published:** 2021-12-11

**Authors:** Katarzyna Mańka-Malara, Maciej Trzaskowski, Dominika Gawlak

**Affiliations:** Department of Prosthodontics, Medical University of Warsaw, 02-097 Warsaw, Poland; maciej.trzaskowski@wum.edu.pl (M.T.); dominika.gawlak@wum.edu.pl (D.G.)

**Keywords:** dental polymers, decontamination, oral hygiene, dental materials, disinfection, dentures, dental prosthesis, oral health, mouth rehabilitation, prosthodontics

## Abstract

Polymeric liners are materials commonly used in prosthodontics to reshape denture surfaces contacting the soft tissues of the oral cavity. The aim of the study was to determine the impact of different cleaning methods on two polymeric materials used in prosthodontics as non-adhesive permanent liners. The material for the research consisted of samples made from Mollosil Plus (Detax, Ettlingen, Germany)—direct polysiloxan liner; and Plastitanium (Pressing Dental, San Marino, Republic of San Marino)—an injection-molded liner. A total of 198 samples were made, 99 of each assessed material. They were exposed to different cleaning methods—a toothbrush, a toothbrush and soap, a toothbrush and toothpaste (BlendaMed, Procter&Gamble, Cincinnati, OH, USA), a toothpaste and denture cleaning paste (Protefix Hygiene Denture Paste, Queisser Pharma, Germany), denture cleansing tablets (Protefix Hygiene Cleaning Tablets, Queisse Pharma, Germany), and a disinfecting spray (Aftermat, Port Jefferson Station, New York City, NY, USA)—for 1 min, 5 min, 10 min, and 15 min. The image acquisition was performed with scanning electron microscopy and samples were analyzed for the homogeneity of their surfaces—the presence of holes, grooves, precipitate, and small and large separating pieces of the material marking departures from this homogeneity. For each type of damage, one point was given. Continuous data from two groups were compared with Mann–Whitney U testing. Due to a small sample size and distribution of variables other than normal, to compare more than two groups, Kruskal–Wallis testing with post hoc analysis (Dunn test with Bonferroni correction) was used. Categorical data were compared with the chi-square test and the Fisher’s exact test. The Mollosil Plus material should be decontaminated with the use of a toothbrush or toothbrush with soap, while Plastitanium material should be disinfected. Plastitanium samples are more susceptible to damage during the decontamination procedures than Mollosil Plus.

## 1. Introduction

Polymeric lining materials are commonly used in prosthodontics to reshape the surface of a denture in contact with soft tissues of oral cavity. Liners can be hard—usually made from polymethylmethacrylate; or resilient—from elastic materials absorbing energy, providing even distribution of the functional loads on the denture bearing area and improving patient’s comfort. Their use is recommended in the case of an inefficient prosthetic base (II and IV class according to the Supple classification), the presence of sharp bone prominences, pain from the area of nerves orifices, during the treatment of prosthetic stomatopathy, and in the post-operative dentures [1,2,3]. The use of relined restorations provides even distribution of forces transferred to soft tissues and increase the patient’s comfort. Among flexible liners are acrylic and silicone materials [4,5,6]. Acrylic liners are copolymers of ethyl methacrylate and alcohols, heat-polymerized or cold-cured, with elastic properties provided by external or internal plasticization. They combine very with acrylic dentures due to their chemical structure, but with the loss of the plasticizer, they become brittle and susceptible to damage [4,7,8]. Polysiloxane materials, such as Mollosil Plus, maintain long-term flexibility in the oral cavity environment but are unable to chemically bond with the acrylic base of the restoration and need an adhesive [4,9,10]. An interesting alternative is the Plastitanium material (Pressing Dental, San Marino, Republic of San Marino), used to reline the denture in the form of an additional, mechanically attached base [11]. This vinyl-based polymer with the addition of titanium has high flexibility and low fluid sorption and therefore is a potentially suitable liner [12].

Proper hygiene of prosthetic restoration has an impact on the health of its user [13,14]. Flexible polymeric restorations may change the pH of the saliva, its buffer capacity, and increase the accumulation of dental and denture plaque [15,16]. The adhesion of microorganisms depends on the composition of the relining material and the denture surface [17,18,19]. Liners are more susceptible to microbial colonization than acrylic denture bases [20,21]. Additionally, silicone materials should be used with special caution, because of the risk of colonization with other strains than *Candida albicans* [22]. To provide the antimicrobial effect of the liner, some authors propose the addition of nanoparticles. Kreve et al. [23] proposed the addition of silver nanoparticles (AgNO_3_), providing an effect against *E. faecalis, P.aeruginosa, and C. albicans*. The incorporation of other nanoparticles, such as zinc oxide (ZnO) and titanium dioxide (TiO_2_), has also been discussed in prosthodontics as a modification of the composition of PMMA denture base [24,25]. The abovementioned Plastitanium, a commercially available liner, has incorporated titanium particles.

Mollosil Plus is commonly used in prosthodontics. Its clinical application was already thoroughly examined in the literature. However, new materials such as Plastitanium are appearing on the market. The injection molding technique provides predictable results and homogenous material, the addition of titanium has an antimicrobial effect, but its resistance to cleaning procedures, conducted daily by all patients, has not been previously examined. The null hypothesis was that decontamination procedures do not affect the surface of both examined polymeric liners. The research aimed to evaluate the influence of decontamination procedures on the surfaces of two permanent lining materials.

## 2. Materials and Methods

The samples made from materials used in prosthodontics for permanent denture relining—Mollosil Plus (Detax, Ettlingen, Germany) and Plastitanium (Pressing Dental, San Marino, Republic of San Marino)—were prepared in the form of cuboids 1 cm long, 1 cm wide, and 0.3 cm thick. Mollosil Plus is a silicon-based, cold-curing material. In the first stage, the multiplied size plate was modeled according to producer instructions and cut into final samples using a scalpel after fabrication. To prepare samples from Plastitanium, the plate was modeled using pink wax and placed in the flask complementary to the injection device—Mg-Newpress (Quattro Ti, KW 700, Cislago, Italy). The injection temperature was set as 165 °C, melting time 20 min, cooling time 20 min, ventilation turned on, pressure 4 bar, slow pace of injection. After cooling, the plate was cut into final samples using a scalpel. A total of 198 samples were made, 99 of each assessed material. The applied methodology was based on the previously published research, but the number of compared samples was increased [26].

Samples were cleaned using a toothbrush, a toothbrush and soap, a toothbrush and toothpaste, a toothbrush and a denture cleaning paste, denture cleaning tablets, and a disinfecting spray. The same type of toothbrush was used in each method—with medium hardness of bristles by Prokudent Interdental (Rossman, SDP, Burgwedel Germany). The pH of 1% aqueous solution of used soap (Biały Jeleń, Polena, Ostrzeszów, Poland) was 9–10. The composition of used fluoride toothpaste (BlendaMed, Procter&Gamble, Cincinnati, OH, USA) was: aqua, sorbitol, hydrated silica, sodium lauryl sulfate, cellulose gum, aroma, CI 77891, trisodium phosphate, sodium fluoride, carbomer, sodium saccharin, limonene, eugenol, polysorbate 80. Ingredients of denture cleaning paste (Protefix Hygiene, Cleaning Paste, Queisser Pharma, Flensburg Germany) were purified water, sorbitol, glycerin, silica, sodium dodecylsulfate, xanthan gum, medium-chain trigliceryde, arome, titanium dioxide, methylparaben, ethylparaben, sodium saccharin, propylparaben. Ingredients declared by the manufacturer of the disinfecting spray (Aftermat, Port Jefferson Station, New York City, NY, USA) were certified organic alcohol and organic essential oil blends. The composition of cleaning tablets (Protefix Hygiene, Active Cleaning Tablets, Queisser Pharma, Flensburg, Germany) was potassium caroate, sodium bicarbonate, sodium carbonate, citric acid, sorbitol, VP/VA copolymer, sodium lauryl sulfate, sodium lauryl sulfoacetate, aroma, Cl 73,015 (color).

The samples were covered with technical gold and viewed under the scanning electron microscope and after 1, 5, 10, and 15 min of exposure—4 for each time point and 16 samples for each method. Total exposure time was the same for each method, and all of them were simulating real applications. One operator performed all procedures. Brushing of each sample was carried out in a wet environment as the toothbrush was moistened in clean water before cleaning, using uniform pressure and without interrupting. During exposure time, the application of disinfecting spray was repeated. The solution of disinfecting tablets was not changed during the time of exposure as the manufacturer recommends using it for 10 min or leaving the appliance in one solution all night. The time of exposure exceeded typical time of cleaning done by a patient to verify what would happen to the surface of the material after a few days of regular cleaning. 

Control samples without any cleaning procedures applied—3 for each material—were also evaluated. All photographs were taken at 1000× magnification with 10 kV voltage accelerating the electron beam in the SE spectrum of secondary electrons. The obtained images were analyzed for the homogeneity of the surface. For each observed type of damage—presence of holes, grooves, precipitate, small separating pieces, and big separating pieces—one point was given. The results achieved for different time points were compared to verify whether the exposure time has an impact on the surface changes of the material. That comparison showed that in some cases there are statistically significant differences between the groups thus, to compare the decontamination methods and two tested materials only samples cleaned for 15 min were used. Statistical analysis of the results was carried out using SPSS v.21 (IBM, Chicago, IL, USA). Continuous data were presented as a mean value and standard deviation (SD). Categorical data were presented as a percentage. Continuous data from two groups were compared by the Mann–Whitney U test. Due to the small sample size and distribution of variables other than normal, to compare more than two groups, the Kruskal–Wallis test with the post-hoc analysis (Dunn test with Bonferroni correction) was used. Categorical data were compared by the chi-square test and Fisher’s exact test, *p*-value less than 0.05 was considered statistically significant.

## 3. Results

**Figure 1:**
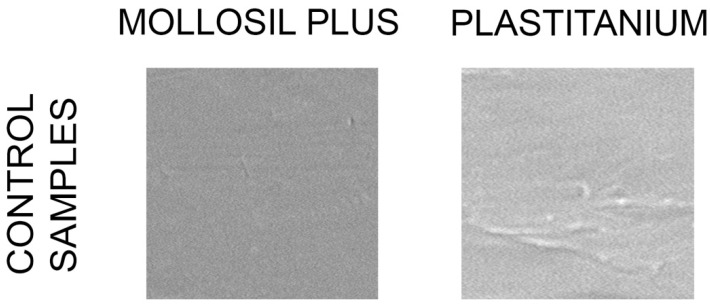
Control samples of tested materials.

**Figure 2:**
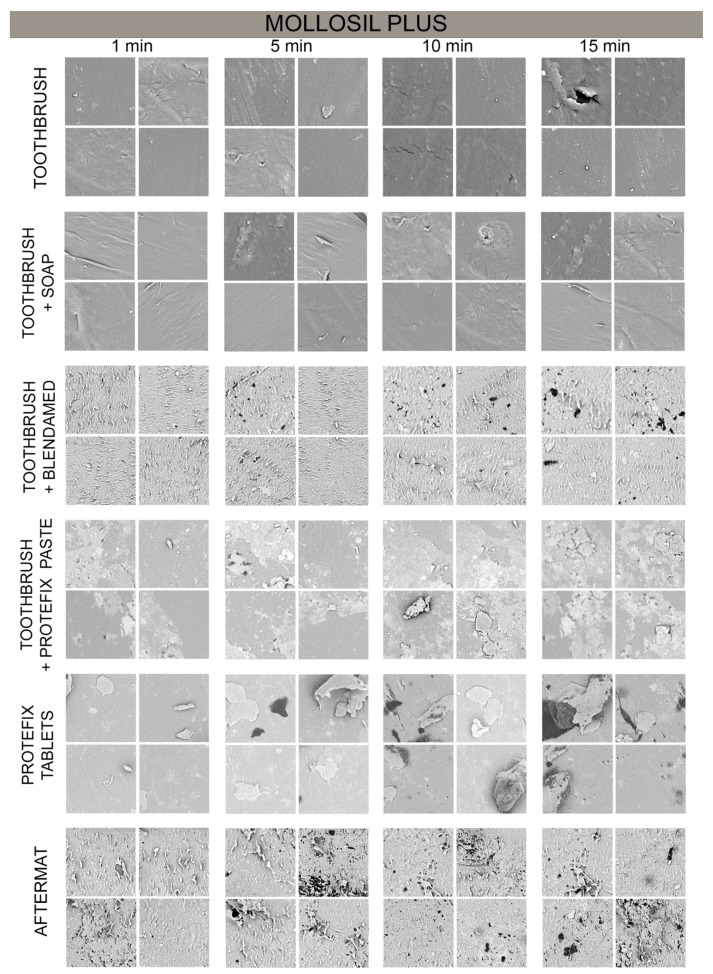
Decontamination of the Mollosil Plus material.

The control samples of tested materials are presented on Figure 1. The Mollosil Plus material was highly homogeneous regardless of the cleaning method used (Figure 2). The use of a toothpaste and a disinfectant spray resulted in the separating of small pieces of the material. In turn, the use of a denture paste left the sediment on the surface (Table 1). In visual evaluation, the most homogenous sample structure was maintained when cleaning with a toothbrush and soap. The post-hoc analysis between the pairs showed statistically significant differences between cleaning with a toothbrush and a toothbrush with Blendamed (*p* = 0.045), a toothbrush and Aftermat (*p* = 0.005), a toothbrush with soap and a toothbrush with Blendamed (*p* = 0.045), a toothbrush with soap and Aftermat (*p* = 0.005), and Protefix tablets and Aftermat (*p* = 0.045).

The time of cleaning had a statistically significant difference only in the case of using Protefix tablets (*p* = 0.005) (Table 2). The post-hoc analysis showed statistically significant differences between the samples between groups decontaminated for 1 min and 5 min (*p* = 0.001), 1 min and 10 min (*p* = 0.017), and 1 min and 15 min (*p* = 0.017).

**Figure 3:**
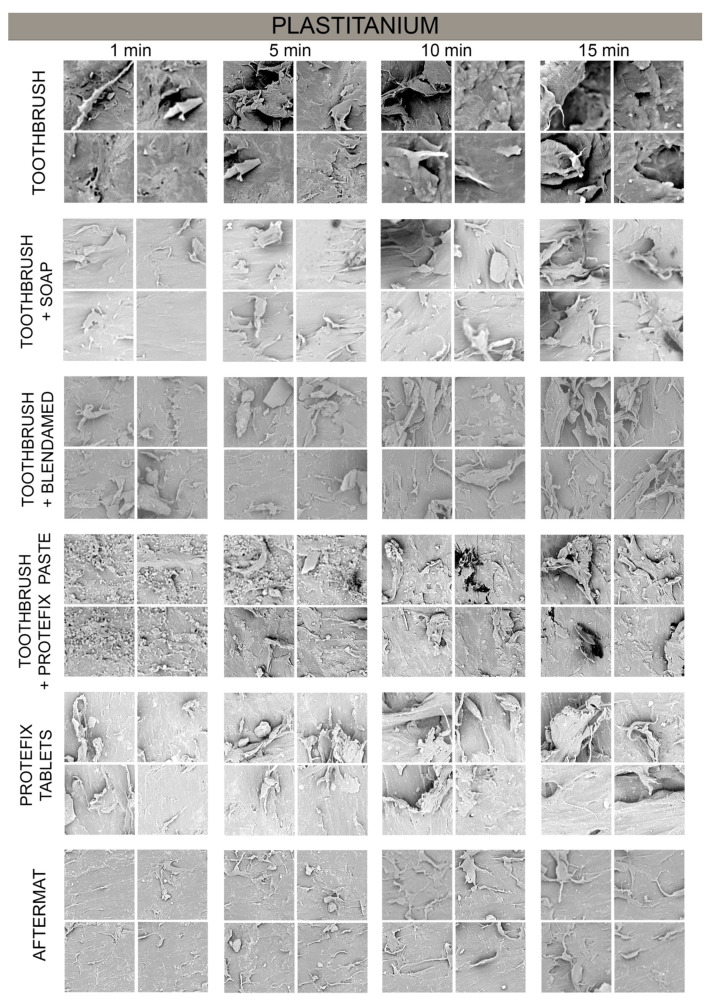
Decontamination of the Plastitanium material.

Plastitanium samples were more susceptible to damage during the decontamination procedures (Figure 3). In the visual evaluation in all samples the change of surface was visible. The smallest fragments were separated after disinfection. Table 3 contains the evaluation of the surface of Plastitanium samples exposed to different decontamination methods. The statistically significant differences (*p* = 0.008) comparing each type of damage were observed only for the presence of precipitate. The comparison of the sums of damage points showed the lowest damage for the use of Aftermat (3.5 ± 0.6) and the highest for Protefix tablets (4.8 ± 0.05). The post-hoc analysis showed statistically significant differences between the pairs: a toothbrush and Aftermat (*p* = 0.030), a toothbrush with soap and a toothbrush with Blendamed (*p* = 0.009), a toothbrush with soap and Protefix tablets (*p* = 0.030), a toothbrush with Blendamed and Aftermat (*p* = 0.002), a toothbrush with Protefix paste and Aftermat (*p* = 0.030), and Protefix tablets and Aftermat (*p* = 0.009).

The statistically significant differences between different times of exposure were observed for samples cleaned using a toothbrush and Protefix paste (*p* = 0.035) (Table 4). The post-hoc analysis between the groups showed statistically significant differences between the Plastitanium samples cleaned for 1 min and 10 min (*p* = 0.020), and 1 min and 15 min (*p* = 0.009). There was also observed such tendency for samples decontaminated with the use of a toothbrush and soap, but it was not statistically significant.

Due to the statistically significant differences depending on the time of exposure (Table 2 and Table 4), when comparing the influence of the decontamination method and materials, we did not use all time-point samples, but only damage points from the samples decontaminated for 15 min. There were statistically significant differences between the Mollosil Plus material and the Plastitanium when comparing the total sum of damage after decontamination (15 min of exposure) using a toothbrush, a toothbrush with soap, a toothbrush and Blendamed, a toothbrush with Protefix paste and Protefix tablets (Table 5). In the above-mentioned comparisons, Mollosil Plus samples were less damaged. Only for disinfection with Aftermat spray were the differences not statistically significant. Due to damage to the sample surface observed in both materials, the null hypothesis that decontamination does not affect the surface of examined polymeric liners could not be accepted.

## 4. Discussion

Lining denture materials, remaining flexible over a long period of use, have many advantages from the clinical point of view. Although they do not chemically adhere to the denture base, the use of additional methods—such as adhesive or mechanical bonding— provides predictable results [4,11]. Unfortunately, the adhesion of microorganisms and possible damage of the surface by bacterial colonization allows only temporary application [27]. The conducted study shows that proper cleaning of elastic polymeric materials is also not easy. Although the evaluated polymers belonged to one type of prosthodontic supplemental materials, their cleaning methods should be different. Polysiloxane liner—Mollosil Plus—should be decontaminated using a toothbrush or toothbrush with soap, just like standard acrylic dentures. Its high resistance to cleaning may possibly be explained because silicone materials are highly hydrophobic, and only a small amount of water absorption and degradation occurs [28]. In our research, brushing was performed in a wet environment. Such conditions resemble actual cleaning performed by a patient. Choosing the same method for Plastitanium material—a vinyl polymer with the addition of titanium—results in significant damage to the surface. The optimal method of cleaning this type of denture lining is disinfection without mechanical friction. This material, due to its significant susceptibility to mechanical damage, should be evaluated in further studies assessing its properties or applied only temporarily. The hardness and durability of this material have not been tested. It could be more susceptible to damage having more elastic properties than the Mollosil Plus material. Additionally, Plastitanium contains the addition of titanium. The manufacturer does not provide the information of the weight fraction nor concentration of particles, which is important as it has an impact on the material mechanical properties [29]. The possible explanation of its higher susceptibility to damage during decontamination could also be its release from the material. Conducted tests are only a simulation of patient cleaning performance, but the results indicate that recommendations concerning cleaning the relined denture should always be selected individually depending on the used material. 

Studies on the effects of cleaning on lining materials have already been conducted, but the different choices of materials make the direct comparison of the results impossible. Many studies concentrate on the properties of lining materials depending on decontamination methods. Mahboub et al. [30] evaluated decontamination of acrylic lining material (Soft liner, GC Corporation, Tokyo Japan) with the Corega disinfecting tablets (GlaxoSmithKline, Brentfort UK), 2.5% sodium hypochlorite, and distilled water (as a control group). They showed that the use of disinfectants resulted in greater resistance to shear and tensile forces, and thus lower the risk of separating the liner from the denture base. The analysis carried by Farzin et al. [31] did not show statistically significant differences in the bond strength of lining material to the acrylic material depending on the purification method used. The material used in this study was acrylic EverSoft (Myerson, Austenal Inc., Chicago, IL, USA) and the cleaners, Fitty dent (Fittydent International GmbH, Austria) and Calgon (The Clorox Co, Oakland, CA, USA). The test of the hardness of the liners (Luci Sof, Dentsply International, Charlotte, NC, USA; Molloplast B, Detax-Gmbh&Co., Ettlingen, Germany; Sofeliner, Tokuyama Dental Corp., Tokyo, Japan), depending on cleaning method (Efferdent, Warner-Lamber, Lynchburg, VA, USA and 0.5% alkaline hypochlorite), showed that they differed significantly from each other, regardless of the exposure time and the agent used. Simulation of the two-year use of cleaning agents did not affect their hardness [32]. Studies evaluating the effect of decontamination products on the hardness and roughness of silicone and acrylic liners revealed that silicon polymers were less prone to damage [33,34]. The use of different materials makes it difficult to compare the results obtained in the literature. Despite the different methodology applied in our study, the polysiloxane liner also showed greater durability to applied cleaning agents. None of the studies carried out in the literature so far have included a new type of material such as Plastitanium. However, the obtained results suggest that, due to high susceptibility to damage, it should not become the first-choice material. Additionally, it is difficult to use clinically—the lining must be prepared separately by thermal injection, and it is kept in place by retention cuts in the denture base [11,12].

Many cleaning agents which can be used for dentures relined with elastic materials were evaluated in the literature [35,36,37]. The main difficulty is that liners are susceptible to change—extrinsic substances may incorporate into the material, loss of plasticizer in resin-based liners may result in degradation [38]. Materials that do not have plasticizers in their composition absorb less water during aging and have less increase in hardness [39,40]. Additionally, the choice of the best disinfectant is difficult because not all manufacturers declare the precise composition of their product. However, in real-life situations, patients choose the commercially available product. Disinfectants containing ethanol—such as the Aftermat spray in the present study—may affect the surface roughness [41,42]. In research concerning polymethyl methacrylate PMMA, chemical disinfecting agents containing alcohol affect flexural strengths of the non-crosslinked denture base resins and affect the interphase region between PMMA polymer bead and the polymer matrix [42,43]. The present study showed that disinfectant with alcohol caused the lowest damage to the surface of Plastitanium samples. Further research would be beneficial to verify whether it does not adversely affect other properties of this material. Other disinfectants commonly used in prosthodontics contain chlorhexidine, glutaraldehyde, sodium hypochlorite, quaternary ammonium compounds (QUATs), alkylamine, or active oxygen. Glutaraldehyde does not have a strong influence on the properties of acrylic or rubber materials and is broadly used [44]. However, it can penetrate the surface of the denture and cause allergic reactions [45]. Raszewski et al. [46] reported also that chlorhexidine disinfecting gel can be successfully used for PMMA denture base disinfection. Other modifications of prosthodontic restoration to increase antimicrobial activity were also discussed. Walczak et al. [47] studied the chitosan coating of PMMA and PETG material due to its antimicrobial and hemostatic activity and the influence of disinfecting its surface. Many studies have also addressed the possibility of introducing antifungal compounds to the liners [48,49,50,51]. 

Patients are often given unified recommendations concerning their prosthetic appliance. Introducing new polymeric material into clinical practice in prosthodontics requires previous verification of all its features. This study included a comparison of only two selected materials that do not connect adhesively to the denture base. Mollosil Plus is commonly used, both in the research as an example of auto-polymerized silicone-based denture lining material, and in clinical use due to its fast and predictable results [52,53,54]. Previous studies concerning elastic polymers used in prosthodontics for mouthgaurds showed that injection-molded materials have advantageous mechanical and clinical properties [55,56,57]. Plasitatnium is a unique pressure-injected prosthodontic liner. Addition of titanium could also possibly provide an antimicrobial effect. However, current research suggests that further studies are needed to verify whether it can be used as a permanent liner and how the disinfection changes the mechanical properties of this material. Due to differences in materials’ characteristics, all polymers introduced into clinical application should be tested to develop recommendations for each of them.

The current study employed times of exposure exceeding typical cleaning times that would be performed by a patient. Our goal was to verify what would happen after few days of regular cleaning as a patient receives a relined restoration for a daily use. We assumed that the time between the exposures can be missed and proposed a prolonged time of cleaning. These conditions do not precisely replicate actual conditions and should be considered as a limitation of this study. However, all cleaning procedures performed during the research were done by one researcher, the time of cleaning was exactly measured, the results of this research are repeatable and thus can compare the effect of decontamination methods on the surface of the material. It should be also stated that comparing such influence is not sufficient to state that declared methods are the best from a microbiological point of view. To get the full view, further research should include the evaluation of the effectiveness of different cleaning methods on each polymeric lining material.

## 5. Conclusions

Under the conditions of this study, it can be concluded that the optimal method of cleaning the Mollosil Plus material, due to the influence on the surface structure, is the use of a toothbrush or a toothbrush with soap and to disinfect the Plastitanium with spray. Mollosil Plus is less prone to surface damage which may occur during cleaning procedures. Further research on the greater range of permanent lining materials would be beneficial to develop recommendations for each type of commercially used material.

## Figures and Tables

**Table 1:** Influence of decontamination methods on Mollosil Plus samples.

Mollosil Plus	1 Toothbrush 15 min (*n* = 4)	2 Toothbrush + Soap 15 min (*n* = 4)	3 Toothbrush + Blendamed 15 min (*n* = 4)	4 Toothbrush + Protefix Paste 15 min (*n* = 4)	5 Protefix Tablets 15 min (*n* = 4)	6 Aftermat 15 min (*n* = 4)	*p*
Small separating pieces, *n* (%)	2 (50.0)	3 (75.0)	4 (100.0)	3 (75.0)	2 (50.0)	4 (100.0)	0.377
Big separating pieces, *n* (%)	1 (25.0)	0 (0.0)	2 (50.0)	4 (100.0)	2 (50.0)	3 (75.0)	0.075
Precipitate, *n* (%)	0 (0.0)	0 (0.0)	4 (100.0)	2 (50.0)	4 (100.0)	4 (100.0)	0.001
Grooves, *n* (%)	2 (50.0)	3 (75.0)	0 (0.0)	0 (0.0)	0 (0.0)	0 (0.0)	0.020
Holes, *n* (%)	1 (25.0)	0 (0.0)	0 (0.0)	0 (0.0)	0 (0.0)	1 (25.0)	0.498
Sum, mean ± SD	1.5 ± 1.0	1.5 ± 0.6	2.5 ± 0.6	2.3 ± 0.5	2.0 ± 0.0	3.0 ± 0.8	0.031 p 1,3 = 0.045 p1,6 = 0.005 p2,3 = 0.045 p2,6 = 0.005 p5,6 = 0.045

**Table 2:** Influence of the decontamination time on Mollosil Plus samples—according to the sum of points for all evaluated types of damage.

Decontamination Method.	1 min (mean ± SD)	5 min (mean ± SD)	10 min (mean ± SD)	15 min (mean ± SD)	*p*
Toothbrush	0.25 ± 0.5	1.25 ± 0.5	1.0 ± 0.8	1.5 ± 1.0	0.147
Toothbrush + soap	1.75 ± 0.5	1.25 ± 1.3	0.75 ± 1.0	1.5 ± 0.6	0.449
Toothbrush + Blendamed	2.25 ± 0.5	2.25 ± 0.5	3.0 ± 0.8	2.5 ± 0.6	0.310
Toothbrush + Protefix paste	1.5 ± 0.6	2.25 ± 0.5	2.25 ± 1.0	2.25 ± 0.5	0.324
Protefix Tablets	1.25 ± 0.5	2.5 ± 0.6	2.0 ± 0.0	2.0 ± 0.0	0.005 p1,2 = 0.001 p1,3 = 0.017 p1,4 = 0.017
Aftermat	2.25 ± 0.5	3.0 ± 0.8	2.5 ± 0.6	3.0 ± 0.8	0.360

**Table 3:** Influence of decontamination methods on the Plastitanium samples.

Plastitanium	1 Toothbrush 15 min (*n* = 4)	2 Toothbrush + Soap 15 min (*n* = 4)	3 Toothbrush + Blendamed 15 min (*n* = 4)	4 Toothbrush + Protefix Paste 15 min (*n* = 4)	5 Protefix Tablets 15 min (*n* = 4)	6 Aftermat 15 min (*n* = 4)	*p*
Small separating pieces, *n* (%)	4 (100.0)	4 (100.0)	4 (100.0)	4 (100.0)	4(100.0)	4 (100.0)	-
Big separating pieces, *n* (%)	4 (100.0)	4 (100.0)	4 (100.0)	4 (100.0)	3 (75.0)	4 (100.0)	0.390
Precipitate, *n* (%)	2 (50.0)	0 (0.0)	4 (100.0)	4 (100.0)	4 (100.0)	3 (75.0)	0.008
Grooves, *n* (%)	4 (100.0)	4 (100.0)	4 (100.0)	3 (75.0)	4 (100.0)	2 (50.0)	0.156
Holes, *n* (%)	4 (100.0)	3 (75.0)	4 (100.0)	3 (75.0)	4 (100.0)	1 (25.0)	0.066
Sum, mean ± SD	4.5 ± 0.6	3.8 ± 0.5	5.0 ± 0.0	4.5 ± 1.0	4.8 ± 0.5	3.5 ± 0.6	0.016 p1,6 = 0.030 p2,3 = 0.009 p2,5 = 0.030 p3,6 = 0.002 p4,6 = 0.030 p5,6 = 0.009

**Table 4:** Influence of the decontamination time on Plastitanium samples—according to the sum of points for all evaluated types of damage.

Decontamination Method	1 min (mean ± SD)	5 min (mean ± SD)	10 min (mean ± SD)	15 min (mean ± SD)	*p*
Toothbrush	4.0 ± 0.8	3.5 ± 0.6	4.3 ± 0.5	4.5 ± 0.6	0.193
Toothbrush + soap	2.8 ± 0.5	3.5 ± 0.6	3.8 ± 0.5	3.8 ± 0.5	0.057
Toothbrush + Blendamed	4.5 ± 0.6	4.8 ± 0.5	4.8 ± 0.5	5.0 ± 0.0	0.517
Toothbrush + Protefix paste	2.8 ± 1.0	3.5 ± 0.6	4.3 ± 0.5	4.5 ± 1.0	0.035 p1,3 = 0.020 p1,4 = 0.009
Protefix Tablets	3.8 ± 1.0	4.8 ± 0.5	4.5 ± 1.0	4.8 ± 0.5	0.269
Aftermat	2.5 ± 0.6	3.0 ± 0.8	3.3 ± 0.5	3.5 ± 0.6	0.193

**Table 5:** Comparison between decontamination of the Molosil Plus and the Plastitanium samples—according to the sum of points for all evaluated types of damage.

Decontamination Method	Plastitanium (mean ± SD)	Molosil (mean ± SD)	*p*
Toothbrush	4.5 ± 0.6	1.5 ± 1.0	0.002
Toothbrush + soap	3.8 ± 0.5	1.5 ± 0.6	0.001
Toothbrush + Blendamed	5.0 ± 0.0	2.5 ± 0.6	0.003
Toothbrush + Protefix Paste	4.5 ± 1.0	2.3 ± 0.5	0.007
Protefix Tablets	4.8 ± 0.5	2.0 ± 0.0	0.002
Aftermat	3.5 ± 0.6	3.0 ± 0.8	0.356
